# Association between statin administration and *Clostridium difficile*-induced enteritis: a retrospective analysis of the MIMIC-IV database

**DOI:** 10.3389/fphar.2025.1550378

**Published:** 2025-02-24

**Authors:** Renli Wang, Rongjun Liu, Hua Wang, Zhaojun Xu

**Affiliations:** ^1^ Department of Intensive Care Unit, Ningbo No. 2 Hospital, Ningbo, Zhejiang, China; ^2^ Department of Anesthesiology, Ningbo No. 2 Hospital, Ningbo, Zhejiang, China

**Keywords:** *Clostridium difficile*-induced enteritis, MIMIC-IV database, intensive care unit, incidence, in-hospital mortality

## Abstract

**Background:**

Existing research suggests that using statins may reduce the incidence of enteritis caused by *C. difficile* and improve the prognosis of patients. This study aimed to explore the relation between *Clostridium difficile*-induced enteritis (CDE) and statin use.

**Methods:**

Data were collected from the Medical Information Mart for Intensive Care-IV (MIMIC-IV) database. Multivariate logistic regression analysis was employed to assess the impact of statin use on CDE incidence in patients in intensive care units (ICUs) and its effect on in-hospital mortality among them. The research findings were validated by performing propensity score matching (PSM), inverse probability of treatment weighting (IPTW), and subgroup analyses.

**Results:**

The study enrolled the data of 51,978 individuals to assess the effect of statin usage on the occurrence of CDE in patients admitted to the ICU. The results indicate that statins can decrease the prevalence of CDE in patients in ICU (odds ratio (OR): 0.758, 95% confidence interval (CI): 0.666–0.873, *P* < 0.05), which was further confirmed through PSM (OR: 0.760, 95% CI: 0.661–0.873, *P* < 0.05) and IPTW (OR: 0.818, 95% CI: 0.754–0.888, *P* < 0.05) analyses. For most subgroups, statins’ favorable effect in reducing CDE remained constant. A total of 1,208 patients were included in the study to evaluate whether statins could lower the risk of death in patients in ICU with enteritis caused by *C. difficile*. Statins did not reduce in-hospital mortality of patients in ICU with CDE (OR: 0.911, 95% CI: 0.667–1.235, *P* = 0.553). The results were validated following PSM (OR: 0.877, 95% CI: 0.599–1.282, *P* = 0.499) and IPTW (OR: 0.781, 95% CI: 0.632–1.062, *P* = 0.071) analyses, and all subgroups demonstrated consistent results.

**Conclusion:**

Statin administration can reduce the incidence of CDE in patients in the ICU; however, it does not decrease the in-hospital mortality rate for individuals with CDE.

## 1 Introduction

*Clostridium difficile*, a spore-forming, anaerobic Gram-positive *bacillus*, is considered a major causative pathogen of intestinal infection in hospitals, particularly in patients in the intensive care unit (ICU) (Lessa et al., 2012). A survey revealed that approximately 500,000 hospitalizations per year in the United States are associated with *Clostridium difficile* infections, accounting for more than one percent of overall hospital admissions (Finn et al., 2021). Among hospitalized patients, *C. difficile*-induced enteritis (CDE) leads to higher medical costs, prolonged hospital stays, and increased mortality (McDonald et al., 2012). In the United States, the annual healthcare costs associated with CDE vary from $1.1 to $3.2 billion, and the typical hospital stay is extended by 3–6 days for patients infected with *C. difficile* during acute care hospitalizations (Dubberke et al., 2008; Kyne et al., 2002; O’Brien et al., 2007). Despite serial prevention and control measures, CDE has not been effectively controlled, with its incidence continuing to increase due to factors such as the aging population, antibiotic abuse, and rising malignant tumors (Bella et al., 2024).

Inhibitors of 3-hydroxy-9-methylglutaryl-coenzyme A reductase (HMGCR), specifically statins like atorvastatin, rosuvastatin, and simvastatin, are widely used as lipid-lowering drugs for atherosclerosis (Ziaeian and Fonarow, 2017). Recent decades have acknowledged the advantages of statins extend beyond their traditional lipid-lowering effects, as they also possess anti-inflammatory and immunomodulatory properties (Ferri et al., 2013). Although statins are primarily approved for cardiovascular use, they have been effective in enhancing outcomes in acute kidney injury, venous thromboembolism, inflammatory bowel disease, infections such as sepsis, autoimmune conditions like systemic lupus erythematosus, and specific malignancies including hepatocellular and gastric carcinomas (Singh and Singh, 2013; Yu et al., 2015; Ungaro et al., 2016; Kunutsor et al., 2017; Zhang et al., 2024; Li et al., 2023). Statins have also been shown in recent animal research to help resolve intracranial hematomas and reduce neuronal damage after cerebral hemorrhage (Liao et al., 2024).

*Clostridium difficile*-induced enteritis is an infectious condition characterized by inflammation and immune response (Bella et al., 2024). Consequently, it can be hypothesized that statins may confer a protective benefit in patients with CDE. Some previous studies have demonstrated that statins diminish the incidence of nosocomial *C. difficile* infections in hospitalized patients; nevertheless, the specific relation necessitates further investigation (Wijarnpreecha et al., 2019).

The data for this investigation were sourced from Medical Information Mart for Intensive Care-IV (MIMIC-IV), a comprehensive database comprising high-quality clinical information on patients admitted to ICUs in medical centers. The present study aimed to examine the correlation between statin use and CDE in patients in the ICU. The research findings will provide additional data for preventing and treating CDE.

## 2 Methods

### 2.1 Database

Data were retrieved from the MIMIC-IV database (version 3.0), which encompasses essential critical care data of patients admitted to the ICUs at Beth Israel Deaconess Medical Center from 2008 to 2022. The information includes patient demographics, vital signs, laboratory tests, administered drugs, and nursing records. The database was accessed and utilized following authorization by one of the authors after successful completion of the Protecting Human Research Participants training provided by the National Institutes of Health (Renli Wang, certification number: 1797679). All patient data in the database are anonymous; therefore, this study did not require informed consent.

### 2.2 Study population

**FIGURE 1:**
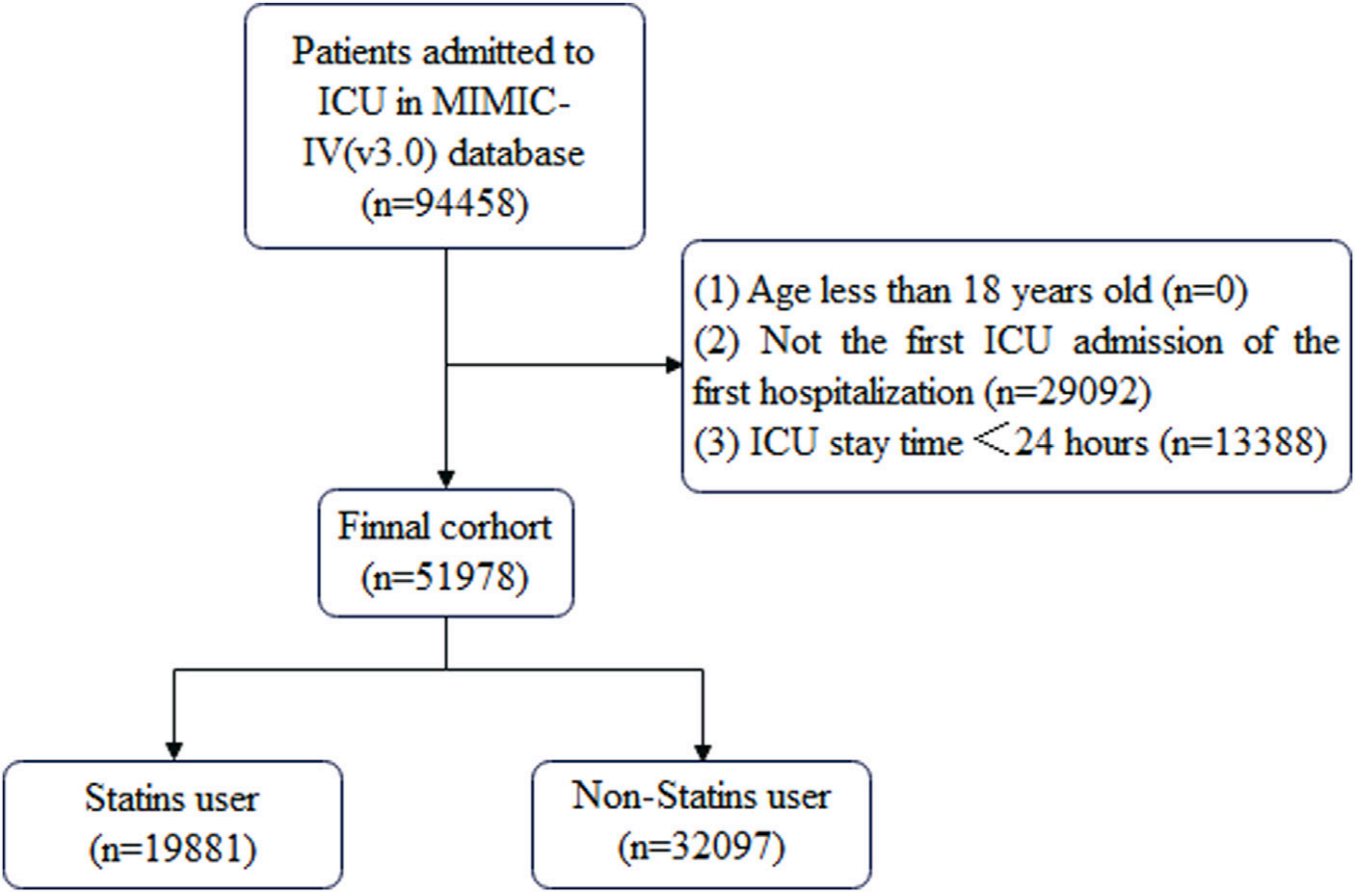
Flowchart depicting the cohort selection procedure.

Patient data that met the following criteria were included in this study: (1) ICU admission during hospitalization, (2) first ICU admission on first hospitalization, (3) ICU stay time ≥24 h, and (4) age ≥18 years. The cohort selection process is shown in Figure 1.

### 2.3 Data extraction

Data were extracted using structured query language (SQL) based on PostgreSQL tools (version 9.6). Patient data were divided into statin and non-statin groups. Data regarding the following covariates were collected: (1) demographic characteristics (age, sex, race, and weight); (2) comorbidities (congestive heart failure, chronic pulmonary disease, severe liver disease, renal disease, cerebrovascular disease, rheumatic disease, cancer, diabetes, and the Charlson comorbidity index); (3) disease severity assessment [sequential organ failure assessment (SOFA) score, simplified acute physiology score II (SAPS II), renal replacement therapy (RRT), and mechanical ventilation (MV)]; (4) drug use [antibiotics (clindamycin, fluoroquinolones, and third-generation cephalosporins) and proton pump inhibitors (PPIs)]; (5) initial vital signs upon ICU admission [heart rate (HR), mean arterial pressure (MAP), respiratory rate (RR), and temperature (°C)]; (6) first laboratory tests result after entering ICU (hemoglobin, platelet counts, white blood cell (WBC) count, prothrombin time (PT), partial thromboplastin time (PPT), blood urea nitrogen (BUN), creatinine, alanine aminotransferase (ALT), aspartate aminotransferase (AST), total bilirubin, glucose, lactate, albumin, and oxygenation index).

### 2.4 Outcomes

The primary outcome was the occurrence of CDE in patients admitted to the ICU, and the secondary outcome was the in-hospital death rate of patients with CDE in the ICU.

### 2.5 Statistical analyses

Variables with >60% missing values were excluded from the analysis. Multiple imputation by weighted predictive mean matching was performed for variables with <60% missing values (Zhang et al., 2019; Hu et al., 2023). The “mice” package in RStudio was employed to impute the data (Zhang et al., 2019). The extent and proportion of missing data for each covariate in the MIMIC-IV are reported in Supplementary Table 1.

Baseline characteristics are presented as mean (standard deviation) or median (interquartile range) for continuous variables and number (percentage) for categorical variables. The chi-squared (χ^2^) test, t-test, or Wilcoxon rank-sum test was used to compare the characteristics of patients in different groups as appropriate.

The primary analysis used in this study was logistic regression. The results are represented as odds ratios (ORs) and coefficients with their respective 95% confidence intervals (95% CIs). The potential of statins to mitigate the incidence of CDE was assessed using five models. Model 1 was unadjusted; Model 2 was adjusted for age, sex, and race; Model 3 was adjusted for the factors in Model 2 and congestive heart failure, chronic pulmonary disease, severe liver disease, renal disease, cerebrovascular disease, rheumatic disease, cancer, diabetes, and Charlson comorbidity index; Model 4 was adjusted for the characteristics in Model 3 and SOFA and SAPS II scores; and Model 5 was adjusted for the factors in Model 4 and antibiotic and PPI use. Furthermore, another five models were developed to assess whether statins could reduce in-hospital mortality from CDE. Model 1 remained unchanged; Model 2 was modified to account for age, sex, race, weight, and Charlson index; Model 3 was modified to account for the factors in Model 2 and HR, MAP, RR, and temperature; Model 4 was modified to account for the factors in Model 3 and SOFA score, SAPS II score, RRT, and MV; and Model 5 was modified to account for the factors in Model 4 and hemoglobin, platelet count, WBC count, PT, PPT, BUN, creatinine, ALT, AST, total bilirubin, glucose, lactate, albumin, and oxygenation index. Multicollinearity in the resulting models was quantified using the variance inflation factor (VIF)—variables with VIF >4 were removed.

Propensity score matching (PSM) and propensity score-based inverse probability of treatment weighting (IPTW) were utilized to ensure the robustness of the findings. The study also calculated the standardized mean differences (SMD) and performed χ^2^ or t-tests before and after matching to examine the effects of PSM and IPTW. An SMD >0.1 for a variable can be considered an imbalance between groups (Zhang et al., 2019).

The statistical analysis was conducted using R (version 4.3.2). A *P*-value <0.05 was considered statistically significant.

## 3 Results

### 3.1 Baseline characteristics

A total of 51,978 patients were included in the study to evaluate the impact of statin use on the incidence of CDE; this included 19,881 patients in the statin-using group and 32,097 in the non-statin-using group. All other features, except rheumatic disease, differed between the two groups (Table 1). The data of 1,208 patients with CDE were enrolled in the study to determine the effect of statin therapy on in-hospital mortality. The statin group differed from the non-statin group in numerous variables, including age, RRT, MV, Charlson index, SOFA score, SAPS II score, WBC, PPT, BUN, creatinine, total bilirubin, albumin, and oxygenation index. The comprehensive information can be found in Table 2.

**TABLE 1:** Baseline characteristics of patients admitted to the ICU included in the analysis.

Variables	All (n = 51,978)	Non-statin (n = 32,097)	Statin (n = 19,881)	*P*-value	SMD
Age (years)	65.39 (16.59)	62.31 (18.11)	70.36 (12.58)	<0.001	0.516
Sex [male, n (%)]	29,622 (57.0)	17,256 (53.8)	12,366 (62.2)	<0.001	0.172
Ethnicity [White, n (%)]	32,075 (61.7)	19,566 (61.0)	12,509 (62.9)	<0.001	0.040
Comorbidities					
Congestive heart failure, n (%)	13,022 (25.1)	6,407 (20.0)	6,615 (33.3)	<0.001	0.305
Chronic pulmonary disease, n (%)	12,011 (23.1)	7,038 (21.9)	4,973 (25.0)	<0.001	0.073
Severe liver disease, n (%)	2,501 (4.8)	2,095 (6.5)	406 (2.0)	<0.001	0.223
Renal disease, n (%)	9,506 (18.3)	4,907 (15.3)	4,599 (23.1)	<0.001	0.200
Cerebrovascular disease, n (%)	9,415 (18.1)	5,246 (16.3)	4,169 (21.0)	<0.001	0.119
Rheumatic disease, n (%)	1,690 (3.3)	1,033 (3.2)	657 (3.3)	0.607	0.005
Cancer, n (%)	6,704 (12.9)	5,047 (15.7)	1,657 (8.3)	<0.001	0.229
Diabetes, n (%)	14,784 (28.4)	7,118 (22.2)	7,666 (38.6)	<0.001	0.362
Charlson comorbidity index	4.83 (2.99)	4.46 (3.10)	5.43 (2.68)	<0.001	0.334
Disease severity score					
SOFA score	4.35 (3.19)	4.21 (3.31)	4.58 (2.89)	<0.001	0.117
SAPS II score	35.25 (13.84)	34.25 (14.49)	36.87 (12.54)	<0.001	0.193
Drug use, n (%)					
Antibiotics	19,390 (37.3)	12,095 (37.7)	7,295 (36.7)	0.024	0.020
PPIs	18,797 (36.2)	10,553 (32.9)	8,244 (41.5)	<0.001	0.178
*Clostridium difficile*-induced enteritis	1,208 (2.3)	801 (2.5)	407 (2.0)	0.001	0.030

SMD, standardized mean differences; SOFA, sequential organ failure assessment; SAPS II, simplified acute physiology score II; PPIs, proton pump inhibitors.

**TABLE 2:** Baseline characteristics of patients admitted to the ICU and developed CDE included in the analysis.

Variables	All	Non-statin	Statin	P Value	SMD
N	1,208	801	407		
Age (y)	67.11 (16.17)	64.65 (17.04)	71.96 (13.03)	<0.001	0.482
Male (%)	567 (46.9)	380 (47.4)	187 (45.9)	0.666	0.030
White (%)	773 (64.0)	512 (3.9)	261 (64.1)	0.994	0.004
Weight (kg)	82.82 (23.75)	82.40 (24.32)	83.64 (22.58)	0.390	0.053
Interventions, n (%)					
RRT use	123 (10.2)	67 (8.4)	56 (13.8)	0.005	0.173
MV use	1,021 (84.5)	654 (81.6)	367 (90.2)	<0.001	0.247
Severity					
Charlson index	5.77 (3.12)	5.41 (3.20)	6.49 (2.82)	<0.001	0.356
SOFA score	5.59 (3.36)	5.44 (3.36)	5.88 (3.34)	0.031	0.131
SAPS II score	41.75 (14.24)	40.87 (14.15)	43.50 (13.67)	0.002	0.187
Vital signs					
HR (bpm)	94.91 (21.91)	96.87 (22.22)	91.04 (20.78)	<0.001	0.271
MAP (mmHg)	79.45 (18.82)	79.61 (18.72)	79.13 (19.04)	0.674	0.026
RR (bpm)	20.66 (6.40)	20.85 (6.57)	20.30 (6.05)	0.158	0.087
Temperature (°C)	36.75 (0.83)	36.77 (0.83)	36.72 (0.84)	0.364	0.055
Laboratory tests					
Hemoglobin (g/dL)	10.23 (2.22)	10.16 (2.23)	10.37 (2.22)	0.125	0.094
Platelets ( ×10^9^/L)	219.21 (139.39)	221.87 (149.63)	213.98 (116.62)	0.353	0.059
WBC ( ×10^9^/L)	14.75 (11.09)	15.27 (12.25)	13.71 (18.27)	0.021	0.149
PT (s)	16.90 (9.09)	16.65 (6.84)	17.39 (12.39)	0.183	0.074
PPT (s)	38.13 (21.63)	36.74 (19.25)	40.85 (25.47)	0.002	0.182
BUN (mg/dL)	32.32 (27.22)	30.50 (26.00)	35.91 (29.17)	0.001	0.196
Creatinine (mg/dL)	1.83 (2.22)	1.65 (2.03)	2.17 (2.52)	<0.001	0.229
ALT (U/L)	90.47 (440.40)	93.27 (464.42)	84.97 (389.31)	0.757	0.019
AST (U/L)	142.45 (681.70)	160.15 (767.06)	107.60 (469.40)	0.206	0.083
Total bilirubin (mg/dL)	1.77 (4.19)	2.16 (4.95)	1.01 (1.75)	<0.001	0.308
Glucose (mg/dL)	144.71 (81.37)	141.80 (76.96)	150.42 (89.23)	0.082	0.103
Lactate (mmol/L)	2.17 (1.83)	2.19 (1.69)	2.12 (2.07)	0.510	0.039
Albumin (g/dL)	2.80 (0.62)	2.76 (0.62)	2.87 (0.62)	0.003	0.184
Oxygenation index	243.72 (160.07)	257.09 (163.26)	217.43 (150.38)	<0.001	0.253
In-hospital mortality (%)	228 (18.9)	155 (19.4)	73 (17.9)	0.606	0.036

SMD, standardized mean differences; RRT, renal replacement therapy; MV, mechanical ventilation; SOFA, sequential organ failure assessment; SAPS II, Simplified Acute Physiology Score II; HR, heart rate; MAP, mean arterial pressure; RR, respiratory rate; WBC, white blood cell; PT, prothrombin time; PPT, partial thromboplastin time; BUN, blood urea nitrogen; ALT, alamine aminotransferase; AST, aspartate aminotransferase.

### 3.2 Impact of statin administration on the occurrence of CDE

CDE was diagnosed in 801 (2.5%) and 407 (2.0%) patients in the non-statin and statin-using groups, respectively (Table 1). The unadjusted model indicates that statin utilization may decrease the occurrence of CDE in patients admitted to the ICU (Model 1: OR:0.816, 95% CI: 0.723–0.921, *P* < 0.05) (Table 3). The impact of statin on decreasing the occurrence of CDE persisted even after controlling for several confounding variables (Model 5: OR: 0.758, 95% CI: 0.666–0.873, *P* < 0.05) (Table 3). The estimation bias resulting from the unequal variables across several treatment groups was reduced by employing PSM and IPTW approaches. The variable disparity between groups was markedly reduced following both matches (Supplementary Table 2; Supplementary Figure 1). The outcomes were analogous following PSM (OR: 0.760, 95% CI: 0.661–0.873, *P* < 0.05) and IPTW (OR: 0.818, 95% CI: 0.754–0.888, *P* < 0.05) (Table 3).

**TABLE 3:** Statin administration and CDE incidence.

Model		No. of patients	Or (95% CI)	*P*-value
Unmatched	Model 1	51,978	0.816 (0.723–0.921)	<0.05
	Model 2		0.780 (0.688–0.882)	<0.05
	Model 3		0.794 (0.699–0.902)	<0.05
	Model 4		0.797 (0.701–0.905)	<0.05
	Model 5		0.758 (0.666–0.873)	<0.05
PSM		35,824	0.760 (0.661–0.873)	<0.05
IPTW		103,039.7	0.818 (0.754–0.888)	<0.05

Model 1 was unadjusted. Model 2 was adjusted for age, sex, and race. Model 3 was adjusted for age, sex, race, congestive heart failure, chronic pulmonary disease, severe liver disease, renal disease, cerebrovascular disease, rheumatic disease, cancer, diabetes, and Charlson comorbidity index. Model 4 was adjusted for age, sex, race, congestive heart failure, chronic pulmonary disease, severe liver disease, renal disease, cerebrovascular disease, rheumatic disease, cancer, diabetes, Charlson comorbidity index, SOFA, score, and SAPS II, score. Model 5 was adjusted for age, sex, race, congestive heart failure, chronic pulmonary disease, severe liver disease, renal disease, cerebrovascular disease, rheumatic disease, cancer, diabetes, Charlson comorbidity index, SOFA, score, SAPS II, score, antibiotics use, and PPIs, use.

### 3.3 Effect of statin use on in-hospital mortality in patients with CDE

In-hospital death occurred in 155 patients (19.4%) with CDE who did not use statins and 73 (17.9%) who utilized statins (Table 2). Statin usage did not reduce in-hospital mortality in patients with CDE, either in the adjusted model (Model 5: OR: 0.799, 95% CI: 0.553–1.145, *P* = 0.225) or uncorrected model (Model 1: OR: 0.911, 95% CI: 0.667–1.235, *P* = 0.553) for covariates (Table 4). The matching effect of PSM and IPTW was optimal (Supplementary Table 3; Supplementary Figure 2). The results after PSM (OR: 0.877, 95% CI: 0.599–1.282, *P* = 0.499) and IPTW (OR: 0.781, 95% CI: 0.632–1.062, *P* = 0.071) were comparable to those before matching (Table 4).

**TABLE 4:** The effect of statin use on the in-hospital mortality rate in patients with CDE.

Model		No. of patients	Or (95% CI)	*P*-value
Unmatched	Model 1	1,208	0.911 (0.667–1.235)	0.553
	Model 2		0.717 (0.517–0.986)	0.043
	Model 3		0.764 (0.547–1.057)	0.108
	Model 4		0.858 (0.598–1.221)	0.397
	Model 5		0.799 (0.553–1.145)	0.225
PSM		718	0.877 (0.599–1.282)	0.499
IPTW		2418.4	0.781 (0.632–1.062)	0.071

Model 1 was unadjusted. Model 2 was adjusted for age, sex, weight, race, and Charlson score. Model 3 was adjusted for age, sex, weight, race, Charlson score, HR, MAP, RR, and temperature. Model 4 was adjusted for age, sex, weight, race, Charlson score, HR, MAP, RR, temperature; SOFA, score, SAPS II, score; RRT, and MV., Model 5 was adjusted for age, sex, weight, race, Charlson score, HR, MAP, RR, temperature; SOFA, score, SAPS II, score, RRT, MV, hemoglobin, platelet count; WBC, count, PT, PPT, BUN, creatinine, ALT, AST, total bilirubin, glucose, lactate, albumin, and oxygenation index.

### 3.4 Subgroup analysis

**FIGURE 2:**
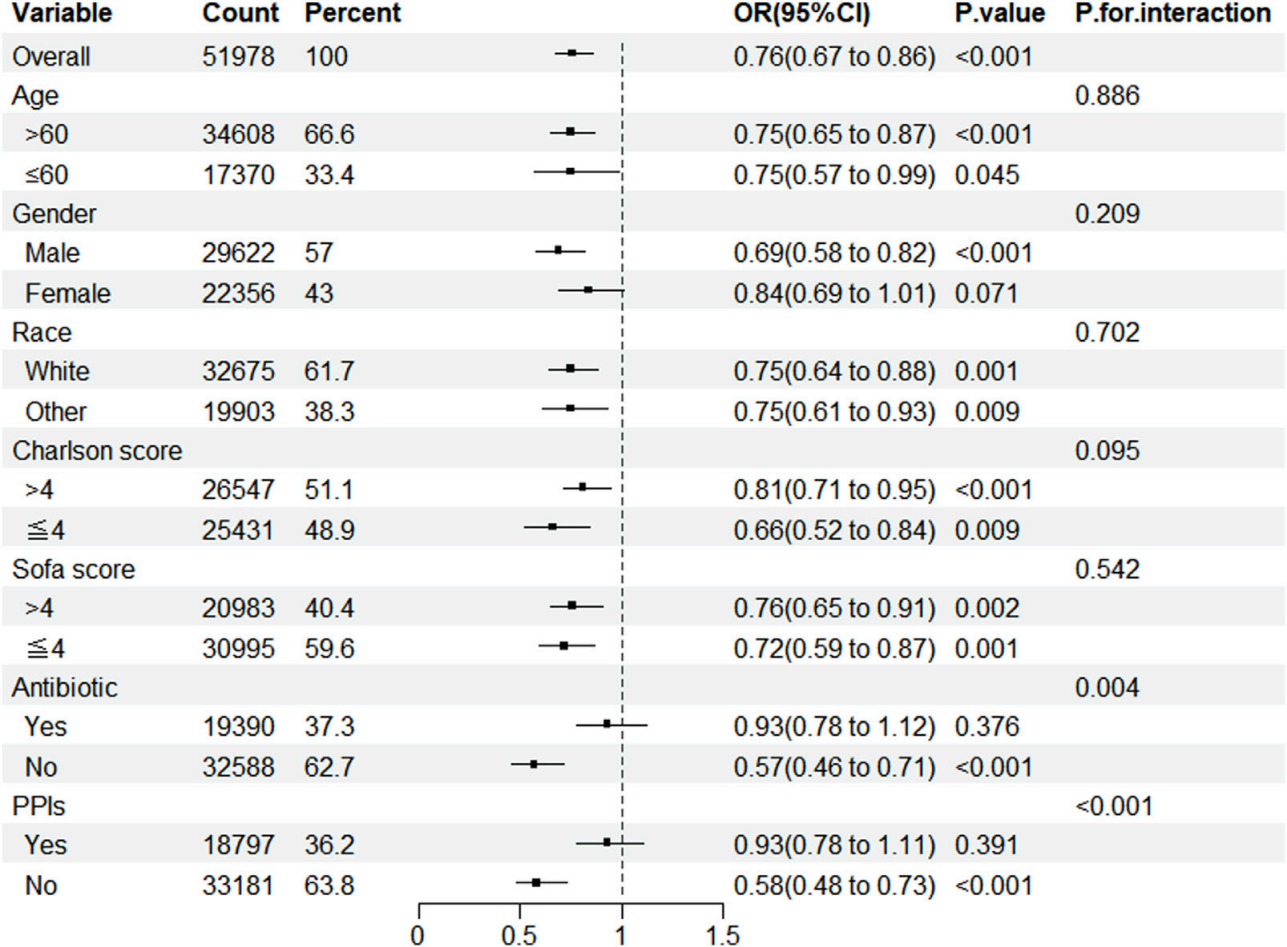
Subgroup analysis of the impact of statin use on the incidence of CDE. Note: ORs (95% CIs) were derived from logistic regression models. Covariates were adjusted as in the model 5. SOFA, sequential organ failure assessment; PPIs, proton pump inhibitors.

Subgroup analysis revealed that, except for female patients and those utilizing antibiotics and PPIs, statin administration lowered the prevalence of CDE in patients admitted to the ICU. Simultaneously, notable interactions were identified in the subgroups categorized by antibiotics and PPIs (Figure 2).

**FIGURE 3:**
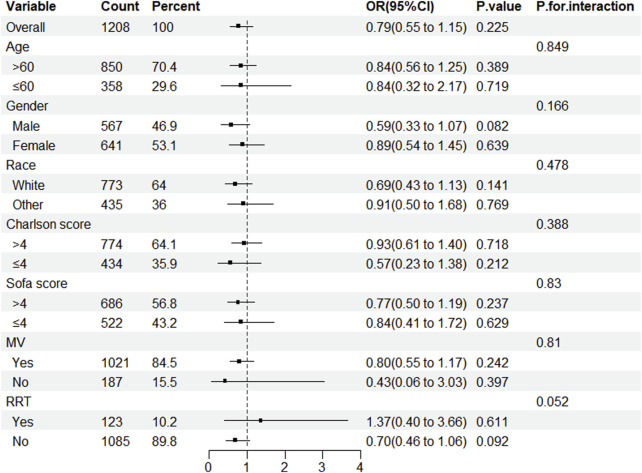
Subgroup analysis of the impact of statin use on in-hospital mortality in patients with CDE. Note: ORs (95% CIs) were derived from logistic regression models. Covariates were adjusted as in the model 5. SOFA, sequential organ failure assessment; MV, mechanical ventilation; RRT, renal replacement therapy.

After conducting a subgroup analysis to determine whether statin usage decreases in-hospital mortality among patients in the ICU with CDE, this study concluded that statins did not reduce in-hospital mortality across any subgroup. No interactions were observed under any strata (Figure 3).

## 4 Discussion

The conditions of patients admitted to the ICU are critical and complex, as they are exposed to antimicrobial medicines and PPIs on a regular and extended basis. This leads to an increased prevalence of CDE in such patients. Karanika S et al. studied 80,835 patients admitted to the ICU and discovered that the infection rate of *C. difficile* in these patients was 2% (95% CI: 1%–2%) (Karanika et al., 2015), similar to the present study’s findings. This study discovered that the incidence rate of CDE in patients in the ICU was 2.32% (1,208/51,978). CDE is, therefore, common among patients in the ICU, and severe infections can lead to septic shock, dilated megacolon, intestinal perforation, and other complications (Bartlett and Gerding, 2008). Consequently, the patients must be provided with adequate clinical care. The existing prevention and treatment strategies for CDE are ineffective; therefore, it is imperative to investigate novel approaches (Guery et al., 2020). Statins are cost-effective and easily accessible. If their involvement in preventing and treating CDE can be established, it would have substantial clinical consequences. Despite the low occurrence, statin usage may lead to adverse effects like aberrant liver function, altered blood sugar levels, and rhabdomyolysis, which warrant attention (Ziaeian and Fonarow, 2017).

Kumarappa VS et al. reported that statin utilization reduced the incidence of *C. difficile* infection among hospitalized patients, although it did not mitigate the risk of such infections in patients outside of hospital settings (Kumarappa et al., 2012). Likewise, Motzkus-Feagans CA et al. documented that statin administration markedly decreased the incidence of *C. difficile* infections in hospitalized patients; however, other lipid-lowering medications did not exhibit this effect (Motzkus-Feagans et al., 2012). Nevertheless, some studies indicate that statin treatment does not decrease the incidence of *C. difficile* infection in patients (Ewelukwa et al., 2014), and some even assert that statins may elevate the risk of such infections (Dobesh et al., 2009). There exist conflicting opinions regarding the impact of statin use on the prognosis of patients with CDE. Park SW et al. found that statin use was associated with a lower in-hospital mortality rate in patients with *C. difficile* infection (Park et al., 2013). On the other hand, Atamna A et al. opined that statin use did not affect the in-hospital mortality rate of patients with CDE (Atamna et al., 2016). Consequently, disagreement persists over the efficacy of statins in lowering the prevalence of CDE and enhancing the prognosis of affected individuals.

The present study found that statin administration during ICU admission may reduce the occurrence of CDE in patients admitted to the ICU; however, statins did not reduce the in-hospital mortality rate among such patients, consistent with findings from several recent studies (Wijarnpreecha et al., 2019). Compared to the other studies, this study was based on the MIMIC database that provides high-quality data and a large sample size; additionally, the study also employed a variety of approaches such as logistical regression, PSM, and IPTW to control potential confounding, enhancing the reliability of the results. This study may also be more therapeutically relevant because it focused on ICUs, where CDE is most common. The mechanisms responsible for the variable effects of statin treatment on the incidence of CDE and the prognosis of people with CDE remain ambiguous. We hypothesize that it may be associated with the following factors: 1) Patients with CDE in the ICU frequently present with additional severe comorbidities, which may directly contribute to mortality; 2) The sample size utilized in examining the effect of statins on the prognosis of CDE patients is very limited. This may potentially impact the credibility of the conclusions to some degree.

The specific mechanisms underlying the reduced incidence of CDE in statin users remain unidentified; however, there exist several plausible theories. First, the decreased risk of *C. difficile* infection may be partly attributed to the immunomodulatory effects of statins, which enhance phagocytes’ capacity to produce extracellular traps and support neutrophil function (Walton et al., 2016). Second, *in vitro* studies indicate that statins may possess direct antibacterial effects, albeit no study has directly proved the antibacterial benefits against *C. difficile* (Giguère and Tremblay, 2004). Third, a murine study demonstrated that statin usage affects gut microbiota and may alter its composition by modifying the transcription of genes that encode proteins essential for gut homeostasis. This may influence the likelihood of acquiring *C. difficile* infection by competing with the normal intestinal microbiota (Nolan et al., 2017). Lastly, statins can demonstrate anti-inflammatory activity by blocking the mevalonate pathway. The decrease in inflammatory response will reduce the severity of *C. difficile* infections, thereby lowering the clinical instances of CDE (Al-Ani, 2013; Shyamsundar et al., 2009).

Subgroup analysis revealed that among patients utilizing antibiotics and PPIs, statin use did not confer a significant protective effect against the incidence of CDE; additionally, an interactive effect was noted between these two subgroups, suggesting that the concurrent use of these drug classes influences the efficacy of statins in mitigating *C. difficile*-induced intestinal infections. The study hypothesizes that this may be due to the following reasons. 1) The administration of antibiotics and PPIs disrupts the absorption or metabolism of statins, thereby diminishing their efficacy; 2) The potent inducing effect of antibiotics and PPIs on CDE obscures the protective effects of statins. Simultaneously, this study observed that the preventive effect of statins against CDE was not pronounced in the female cohort. The sex disparities are hypothesized to influence the body’s immune response and inflammation during *C. difficile* infection, leading to sex-based variations in the preventive efficacy of statins against CDE (Horn et al., 2023).

It is essential to acknowledge the limitations of the present study. Firstly, the data for this investigation were exclusively sourced from the MIMIC-IV database, predominantly comprising patients of White ethnicity, and there may be unobserved confounding variables. Divergences in the gut microbiota among various ethnic groups may influence the efficacy of statins in the prevention of *C. difficile* infection (Gaulke and Sharpton, 2018). Consequently, pertinent studies including various ethnic groups are necessary to validate the generalizability of our research findings. Secondly, not all necessary information is accessible in the MIMIC-IV database. The study’s conclusions may be influenced by the ambiguity surrounding the criteria for statin administration in patients in the critical care units, as some individuals may receive statins post-CDE due to the indeterminate timing of diagnosis. Thirdly, despite the efforts to mitigate the bias by PSM, IPTW, multivariable adjustment, and comprehensive subgroup analysis, the estimation bias was inevitable in this retrospective investigation due to intricate confounding factors in actual clinical treatment that could not be accounted for. Fourthly, the sample size of patients with CDE included in the analysis was limited, potentially influencing the evaluation of the effect of statin use on in-hospital mortality in this patient population to some degree. Fifthly, the study did not assess the influence of various statins and dosages on the prognosis of patients with CDE. Ultimately, the study design did not allow a prolonged investigation; hence, the study could not deduce the effects of statin utilization on long-term outcomes in patients admitted to the ICU with CDE. Further high-quality, large-sample randomized trials are necessary to study the relationship between statin use and CDE to provide more refined guidance for clinical practice.

## 5 Conclusion

Statins can lower the risk of CDE in patients admitted to the ICU, but they do not reduce the in-hospital death rate for such patients. The present research offers a more reliable foundation for administering statins to prevent and treat CDE. However, additional validation is required through forthcoming randomized controlled trials.

## Data Availability

The original contributions presented in the study are included in the article/Supplementary Material, further inquiries can be directed to the corresponding author.
