# Supplementary material for: Association between statin administration and Clostridium difficile-induced enteritis: a retrospective analysis of the MIMIC-IV database
Source: Front Pharmacol. 2025 Feb 24;16:1550378. doi: 10.3389/fphar.2025.1550378 (PMC11891362; doi:10.3389/fphar.2025.1550378)
Supplement: Supplementary file 1 [file DataSheet1.docx]

**Supplementary Table 1** The details on absent data in the pertinent variables.

| **Variables** | **Missing, n (%)** |
| --- | --- |
| Age (y) | 0 (0) |
| Male (%) | 0 (0) |
| White (%) | 0 (0) |
| Weight (kg) | 377 ( 31.20） |
| Interventions, n (%) |  |
| RRT use | 0 (0) |
| MV use | 0 (0) |
| Severity |  |
| Charlson score | 0 (0) |
| SOFA score | 5 (0.41） |
| SAPS II score | 0 (0) |
| Vital signs |  |
| HR (bpm) | 0 (0) |
| MAP (mmHg) | 7 (0.58) |
| RR (bpm) | 0 (0) |
| Temperature (°C) | 2 (0.17） |
| Laboratory tests |  |
| Hemoglobin (g/dl) | 5 (0.41） |
| Platelets (×10^9^/L) | 4 (0.33） |
| WBC (×10^9^/L) | 5 (0.41） |
| PT (s) | 68 (5.63） |
| PPT (s) | 76 (6.29) |
| BUN (mg/dL) | 4 (0.33） |
| Creatinine (mg/dL) | 4 (0.33） |
| ALT (u/l) | 276 (22.85) |
| AST (u/l) | 266 (22.02） |
| Bilirubin total (mg/dL) | 271 (22.43) |
| Glucose (mg/dL) | 4 (0.33） |
| Lactate (mmol/L) | 366 (30.30) |
| Albumin (g/dL) | 431 (35.68) |
| Oxygenation index | 559 (46.27) |

RRT, renal replacement therapy; MV, mechanical ventilation; SOFA, Sequential Organ Failure Assessment; SAPS II, Simplified Acute Physiology Score II; HR, heart rate; MAP, mean arterial pressure; RR, respiratory rate; WBC, white blood cell; PT, prothrombin time; PPT, partial thromboplastin time; BUN, blood urea nitrogen; ALT, alamine aminotransferase; AST, aspartate aminotransferase.

**Supplementary Table 2** Baseline characteristics and SMD of included ICU patients following PSM and IPTW.

| **Variables** | **PSM** | | | | **IPTW** | | | |
| --- | --- | --- | --- | --- | --- | --- | --- | --- |
|  | **Non-Statins** | **Statins** | ***p*** | **SMD** | **Non-Statins** | **Statins** | ***p*** | **SMD** |
| N | 17912 | 17912 |  |  | 52571.2 | 50468.5 |  |  |
| Age (years) | 70.30 (14.82) | 69.54（12.60） | <0.001 | 0.055 | 65.95（17.67） | 67.19（13.79） | <0.001 | 0.078 |
| Gender (male, n [%]) | 10,692（59.7） | 10,897（60.8） | 0.028 | 0.023 | 30,084.2（57.2） | 28,872.7（57.2） | 0.974 | <0.001 |
| Ethnicity (white, n [%]) | 11,287（63.0） | 11,247（62.8） | 0.670 | 0.005 | 32,563.4（61.9） | 31,481.1（62.4） | 0.367 | 0.009 |
| Comorbidities |  |  |  |  |  |  |  |  |
| Congestive heart failure, n (%) | 5,323（29.7） | 5,517 (30.8） | 0.026 | 0.024 | 13,566.5（25.8） | 13,453.3（26.7） | 0.046 | 0.019 |
| Chronic pulmonary disease, n (%) | 4,500 (25.1) | 4,414 (24.6) | 0.299 | 0.011 | 12,265.2 (23.3) | 12,130.5 (24.0) | 0.094 | 0.017 |
| Severe liver disease , n (%) | 332（1.9） | 406（2.3） | 0.007 | 0.029 | 2,510.0（4.8） | 2,672.6（5.3） | 0.081 | 0.024 |
| Renal disease, n (%) | 3,737（20.9） | 3,870（21.6） | 0.088 | 0.018 | 9,837.9（18.7） | 9,835.9（19.5） | 0.045 | 0.020 |
| Cerebrovascular disease, n (%) | 3,654 (20.4) | 3,643 (20.3) | 0.896 | 0.002 | 9,687.5 (18.4) | 9,587.2 (19.0) | 0.135 | 0.015 |
| Rheumatic disease, n (%) | 639 (3.6) | 605 (3.4) | 0.341 | 0.010 | 1,728.4 (3.3) | 1,720.4 (3.4) | 0.503 | 0.007 |
| Cancer, n (%) | 1,634 (9.1) | 1,632 (9.1) | 0.985 | <0.001 | 6,734.7 (12.8) | 6,689.3 (13.3) | 0.238 | 0.013 |
| Diabetes, n (%) | 5,923 (33.1) | 6,284 (35.1) | <0.001 | 0.043 | 15,301.8 (29.1) | 14,948.3 (29.6) | 0.246 | 0.011 |
| Charlson Comorbidity Index | 5.25（2.84） | 5.26（2.68） | 0.757 | 0.003 | 4.90（3.08） | 5.08（2.79） | <0.001 | 0.059 |
| Disease severity score |  |  |  |  |  |  |  |  |
| SOFA score | 4.44（3.20） | 4.51（2.98） | 0.058 | 0.020 | 4.38（3.30） | 4.46（3.09） | 0.019 | 0.025 |
| SAPS II score | 36.83（13.64） | 36.57（12.62） | 0.060 | 0.020 | 35.51（14.28） | 36.25（12.93） | <0.001 | 0.054 |
| Drug use, n (%) |  |  |  |  |  |  |  |  |
| Antibiotics | 6,608（36.9） | 6,552（36.6） | 0.547 | 0.006 | 19,602.8（37.3） | 19,407.1（38.5） | 0.017 | 0.024 |
| PPIs | 6,680（38.4） | 7,025（39.2） | 0.118 | 0.017 | 19,203.2（36.5） | 19,399.6（38.4） | <0.001 | 0.039 |

PSM, Propensity score matching; IPTW, inverse probability of treatment weighing; SMD, standardized mean differences; SOFA, Sequential Organ Failure Assessment; SAPS II, Simplified Acute Physiology Score II; PPIs, proton pump inhibitors.

**Supplementary Table 3** Baseline characteristics and SMD of included ICU patients with CDE after PSM and IPTW.

| Variables | PSM | | | | IPTW | | | |
| --- | --- | --- | --- | --- | --- | --- | --- | --- |
|  | Non-Statins | Statins | *p* | SMD | Non-Statins | Statins | *p* | SMD |
| N | 359 | 359 |  |  | 1,213.6 | 1,204.8 |  |  |
| Age (y) | 70.79 (14.71) | 70.96（13.20） | 0.875 | 0.012 | 67.12（16.61） | 66.20（16.45） | 0.619 | 0.055 |
| Male (%) | 159（44.3） | 166（46.5） | 0.653 | 0.039 | 562.3（46.3） | 533.7（44.3） | 0.611 | 0.041 |
| White (%) | 235（65.5） | 231（64.3） | 0.815 | 0.023 | 775.3（63.9） | 784.2（65.1） | 0.749 | 0.025 |
| Weight (kg) | 83.75 (23.50） | 83.39（22.94） | 0.836 | 0.015 | 82.95（24.23） | 82.79（22.22） | 0.927 | 0.007 |
| Interventions, n (%) |  |  |  |  |  |  |  |  |
| RRT use | 41（11.4） | 45（12.5） | 0.730 | 0.034 | 126.1（10.4） | 170.7（14.2） | 0.321 | 0.115 |
| MV use | 324（90.3） | 320（89.1） | 0713 | 0.037 | 1,029.7（84.4） | 1,064.0（88.3） | 0.155 | 0.102 |
| Severity |  |  |  |  |  |  |  |  |
| Charlson score | 6.34 (3.13) | 6.28 (2.81) | 0.782 | 0.021 | 5.80 (3.24) | 5.71 (2.96) | 0.730 | 0.028 |
| SOFA score | 5.63 (3.22) | 5.78 (3.31) | 0.529 | 0.047 | 5.59 (3.32) | 5.99 (3.78) | 0.405 | 0.112 |
| SAPS II score | 42.41（13.86） | 43.09（13.58） | 0.507 | 0.050 | 41.70 (14.05) | 41.45（13.67） | 0.812 | 0.018 |
| Vital signs |  |  |  |  |  |  |  |  |
| HR (bpm) | 92.49（21.42） | 91.89（20.85） | 0.703 | 0.028 | 94.75（22.26） | 94.88（20.59） | 0.933 | 0.006 |
| MAP (mmHg) | 80.03（19.85） | 79.69（19.09） | 0.815 | 0.017 | 79.46（18.74） | 79.67（18.62） | 0.873 | 0.011 |
| RR (bpm) | 20.85 (6.78) | 20.36 (6.18) | 0.313 | 0.075 | 20.62 (6.58) | 20.68 (6.39) | 0.898 | 0.010 |
| Temperature (°C) | 36.73（0.77） | 36.70（0.85） | 0.559 | 0.044 | 36.74（0.84） | 36.77（0.83） | 0.636 | 0.034 |
| Laboratory tests |  |  |  |  |  |  |  |  |
| Hemoglobin (g/dl) | 10.46（2.19） | 10.33（2.22） | 0.411 | 0.061 | 10.23（2.21） | 10.16（2.23） | 0.700 | 0.031 |
| Platelets (×10^9^/L) | 213.99（124.95） | 212.56（117.07） | 0.874 | 0.012 | 218.89（142.54） | 207.85（125.83） | 0.360 | 0.082 |
| WBC (×10^9^/L) | 13.92（9.34） | 13.77（8.53） | 0.820 | 0.017 | 14.80（11.42） | 15.83（10.83） | 0.349 | 0.092 |
| PT (s) | 16.90（7.58） | 16.24（6.15） | 0.195 | 0.097 | 16.72（7.29） | 18.03（10.98） | 0.327 | 0.140 |
| PPT (s) | 39.34（23.41） | 39.44（22.99） | 0.953 | 0.004 | 38.46（22.15） | 40.10（22.39） | 0.442 | 0.074 |
| BUN (mg/dL) | 35.24（26.98） | 36.30（30.18） | 0.621 | 0.037 | 32.77（27.69） | 32.91（26.72） | 0.937 | 0.005 |
| Creatinine (mg/dL) | 2.12（2.66） | 2.15（2.60） | 0.866 | 0.013 | 1.84（2.31） | 1.93（2.16） | 0.624 | 0.038 |
| ALT (u/l) | 79.02（421.16） | 75.24（388.00） | 0.901 | 0.009 | 94.45（463.31） | 73.46（331.43） | 0.386 | 0.052 |
| AST (u/l) | 110.50（470.92） | 101.66（470.27） | 0.801 | 0.019 | 143.95（679.31） | 108.19（405.97） | 0.228 | 0.064 |
| Bilirubin total (mg/dL) | 1.09（1.47） | 1.03（1.83） | 0.632 | 0.036 | 1.77（4.13） | 2.19（5.08） | 0.671 | 0.090 |
| Glucose (mg/dL) | 148.35（91.74） | 148.62（87.43） | 0.968 | 0.003 | 145.01（88.06） | 144.34（79.68） | 0.905 | 0.008 |
| Lactate (mmol/L) | 2.05（1.32） | 2.07（1.98） | 0.866 | 0.013 | 2.17（1.63） | 2.16（1.98） | 0.897 | 0.009 |
| Albumin (g/dL) | 2.86（0.61） | 2.83（0.61） | 0.464 | 0.055 | 2.81（0.61） | 2.82（0.60） | 0.931 | 0.007 |
| Oxygenation index | 222.46（136.94） | 225.01（155.44） | 0.816 | 0.017 | 244.64（155.72） | 240.08（172.38） | 0.717 | 0.028 |

PSM, Propensity score matching; IPTW, inverse probability of treatment weighing; SMD, standardized mean differences; RRT, renal replacement therapy; MV, mechanical ventilation; SOFA, Sequential Organ Failure Assessment; SAPS II, Simplified Acute Physiology Score II; HR, heart rate; MAP, mean arterial pressure; RR, respiratory rate; WBC, white blood cell; PT, prothrombin time; PPT, partial thromboplastin time; BUN, blood urea nitrogen; ALT, alamine aminotransferase; AST, aspartate aminotransferase.


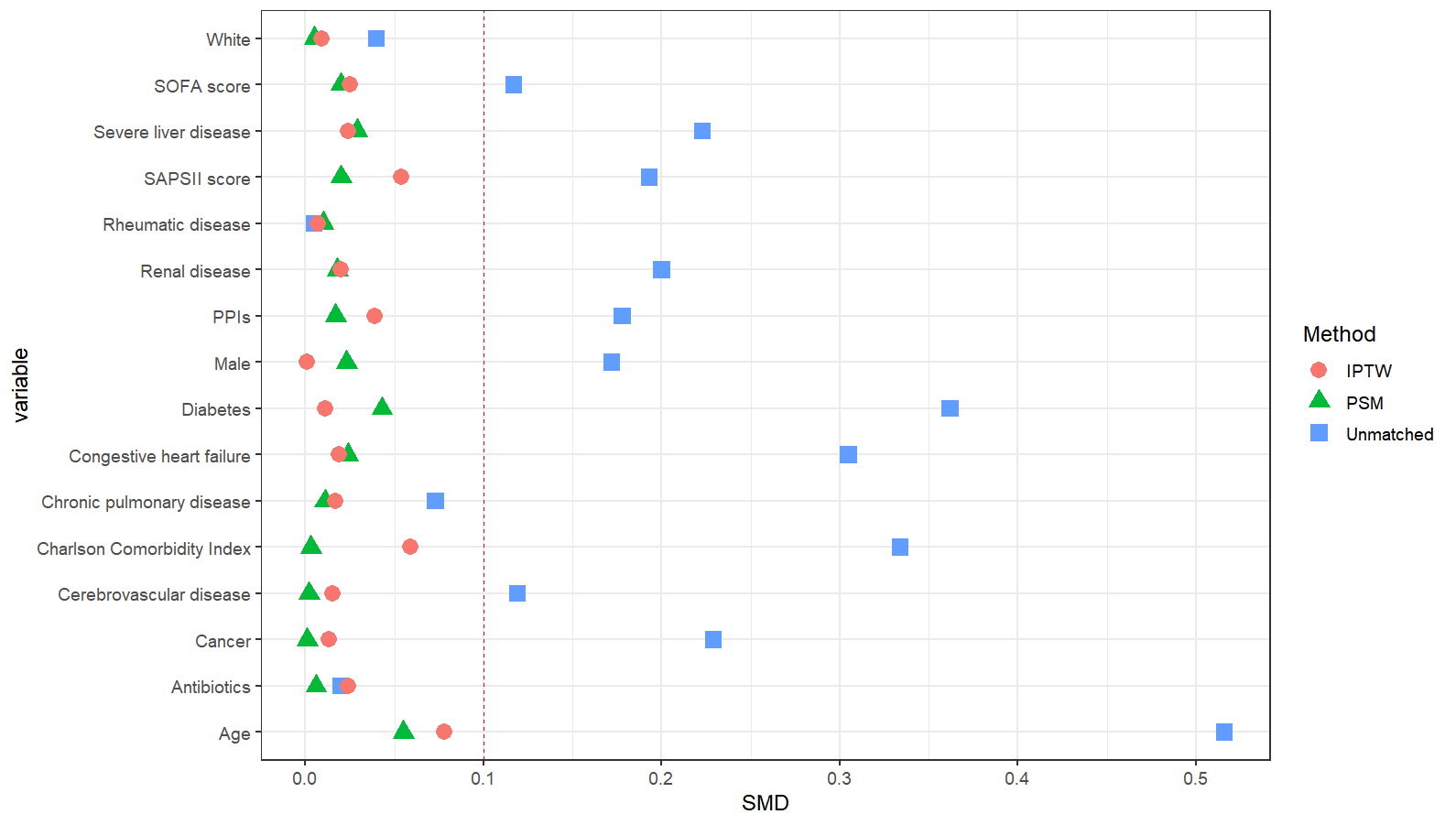


**Supplementary Figure 1** Love plot of balance in baseline characteristics before and after PSM and IPTW of the patients admitted to the ICU included in the analysis. SOFA, sequential organ failure assessment; SAPS II, simplified acute physiology score II; PPIs, proton pump inhibitors.


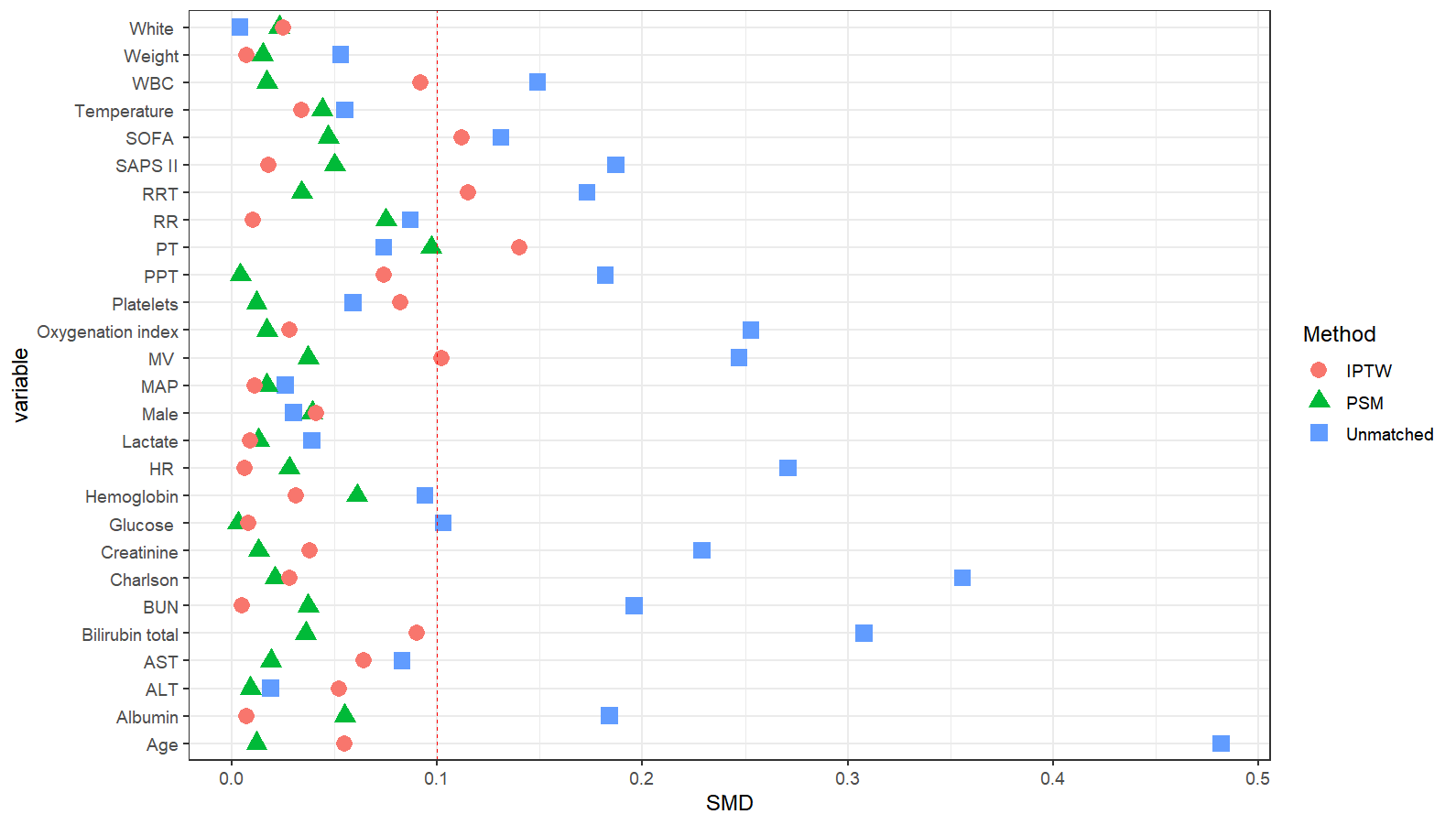


**Supplementary Figure 2** Love plot of balance in baseline characteristics before and after PSM and IPTW of the patients admitted to the ICU and developed CDE included in the analysis. RRT, renal replacement therapy; MV, mechanical ventilation; SOFA, Sequential Organ Failure Assessment; SAPS II, Simplified Acute Physiology Score II; HR, heart rate; MAP, mean arterial pressure; RR, respiratory rate; WBC, white blood cell; PT, prothrombin time; PPT, partial thromboplastin time; BUN, blood urea nitrogen; ALT, alamine aminotransferase; AST, aspartate aminotransferase.
